# Prevalence and Genomic Sequence Analysis of Domestic Cat Hepadnavirus in the United States

**DOI:** 10.3390/v14102091

**Published:** 2022-09-21

**Authors:** Cassidy Stone, Raegan Petch, Roderick B. Gagne, Mary Nehring, Thomas Tu, Julia A. Beatty, Sue VandeWoude

**Affiliations:** 1 Department of Microbiology, Immunology, and Pathology, Colorado State University, Fort Collins, CO 80523, USA; 2 Wildlife Futures Program, Department of Pathobiology, School of Veterinary Medicine, University of Pennsylvania, Philadelphia, PA 19104, USA; 3 Storr Liver Centre, The Westmead Institute for Medical Research, Westmead Hospital, The University of Sydney, Sydney 2145, Australia; 4 University of Sydney Institute for Infectious Diseases, University of Sydney, Sydney 2006, Australia; 5 Centre for Animal Health and Welfare, Department of Veterinary Clinical Sciences, Jockey Club College of Veterinary Medicine and Life Sciences, City University of Hong Kong, Kowloon Tong, Hong Kong, China

**Keywords:** hepadnavirus, hepatitis B virus, immunosuppression, prevalence

## Abstract

Hepadnaviruses are partially double-stranded DNA viruses that infect a variety of species. The prototypical virus in this family is the human hepatitis B virus, which chronically infects approximately 400 million people worldwide and is a risk factor for progressive liver disease and liver cancer. The first hepadnavirus isolated from carnivores was a domestic cat hepadnavirus (DCH), initially identified in Australia and subsequently detected in cats in Europe and Asia. As with all characterized hepadnaviruses so far, DCH infection has been associated with hepatic disease in its host. Prevalence of this infection in the United States has not been explored broadly. Thus, we utilized conventional and quantitative PCR to screen several populations of domestic cats to estimate DCH prevalence in the United States. We detected DCH DNA in 1 out of 496 animals (0.2%) in the U.S. cohort. In contrast, we detected circulating DCH DNA in 7 positive animals from a cohort of 67 domestic cats from Australia (10.4%), consistent with previous studies. The complete consensus genome of the U.S. DCH isolate was sequenced by Sanger sequencing with overlapping PCR products. An in-frame deletion of 157 bp was identified in the N-terminus of the core open reading frame. The deletion begins at the direct repeat 1 sequence (i.e., the 5′ end of the expected double-stranded linear DNA form), consistent with covalently closed circular DNA resultant from illegitimate recombination described in other hepadnaviruses. Comparative genome sequence analysis indicated that the closest described relatives of the U.S. DCH isolate are those previously isolated in Italy. Motif analysis supports DCH using NTCP as an entry receptor, similar to human HBV. Our work indicates that chronic DCH prevalence in the U.S. is likely low compared to other countries.

## 1. Introduction

The *Hepadnaviridae* family consists of partially double-stranded circular DNA viruses that are most notable for establishing hepatic infection and lead to hepatitis [1,2,3,4,5]. The prototypic member of this family is the human hepatitis B virus (HBV), which chronically infects 400 million people world-wide and is associated with approximately 80% of human hepatocellular carcinomas (HCC) [5,6]. While many HBV infections are cleared within 6 months with limited long-term sequelae [4,7], a proportion of infections (approximately 90% of exposures in neonates or children) result in a life-long chronic infection. Chronic infections drive chronic hepatitis, progressive liver injury, and increased risk of HCC [3,4,5,6,7,8].

Manifestations of chronic infections from hepadnaviruses seem to be highly dependent on the species and individual ability to initiate viral clearance. Other hepadnaviruses have been described in woodchucks, ground squirrels, bats, domestic ducks, swine, and geese [9]. Woodchucks have high rates of liver cancer associated with a chronic infection [6,10]. Ground squirrels and bats rapidly clear infection and experience limited liver disease [6,10,11]. Ducks chronically infected with duck Hepatitis B virus can develop liver inflammation, fibrosis, and rarely HCC [12,13,14]. The serum of snow leopards with chronic hepatitis and cholangiohepatitis were shown to cross-react with human HBV surface antigens (suggesting a hepadnavirus infection), but the etiological agent could not be isolated [15].

A novel hepadnavirus was isolated from a domestic cat in Australia in 2016, and designated as domestic cat hepadnavirus (DCH) [1]. DCH is the first hepadnavirus definitively identified in a carnivore [1]. Further studies of DCH have found 10–17.8% rate of DCH DNA positivity in cats infected with feline immunodeficiency virus and/or feline leukemia virus [1,16]. This indicates that coinfection with an immunosuppressive virus may predispose cats to chronic DCH infection, reflecting similar associations with human HBV–HIV co-infection. In another survey conducted in Italy, 10.8% of sampled cats were DCH DNA positive [16]. Finally, DCH DNA was detected in 28% of cats diagnosed with HCC and 43% of chronic hepatitis cases from a combined cohort from the United States and the UK [17,18]. These findings suggest possible pathological effects of DCH in cats and warrant further investigation of the prevalence of the virus in cat populations globally to determine if (1) associations with liver disease are related to high prevalence in the population; and, (2) to estimate the total disease burden associated with DCH. 

In order to further define DCH prevalence in North America, we screened 496 domestic cat samples from privately owned and shelter populations in the U.S. as well as 67 cats from an Australian cohort for circulating and intrahepatic DCH DNA. We identified only one positive individual in the U.S. samples and describe a unique genomic deletion in the genetic sequence from this isolate.

## 2. Materials and Methods

### 2.1. Sample Collection

DNA extracted from domestic cat whole blood samples that were archived from previous studies were screened for DCH DNA. This included 286 samples from shelter animals in California, Colorado, and Florida [19]; 57 samples from a multi-cat household with FeLV infection in Maryland [20]; 84 clinical samples from cats with hepatic lymphoma from across the U.S. (gift of Anne C. Avery, CSU Clinical Hematopathology Laboratory); 58 samples of domestic cats with FIV infection from U.S. shelters from Tennessee [21]; and 67 samples from a hospital population in Australia [22]. Eleven liver samples from cats with no history of hepatic disease were obtained from domestic cats that presented to Colorado State University for necropsy and were donated by owners for educational purposes. Sample details are detailed in Table 1.

### 2.2. DNA Isolation

Tissue DNA Extraction: Liver tissue samples were snap frozen and stored at −80 °C. They were then trimmed to 20 mg and placed in a lysing matrix D tube (MP Biomedicals, Fisher Scientific, Waltham, MA, USA). DNA was extracted using QIAGEN DNeasy blood and tissue kit (QIAGEN Inc., Valencia, CA, USA). Briefly, 450 µL buffer ATL and 50 µL proteinase K were added to the lysis tube with the tissue. Samples were homogenized for 60 s in FastPrep^®^ centrifuge. Tubes were centrifuged at 14,000× *g* for 10 min to pellet debris. 200 µL supernatant was transferred into a 1.7 mL tube. Final elution was performed with Qiagen EB buffer (10 mM Tris-Cl) instead of AE buffer (10 mM Tris-Cl, 0.5 mM EDTA) (QIAGEN, Inc., Valencia, CA, USA). 

Plasma DNA Extraction: Plasma DNA was extracted using the QIAamp UCP Pathogen Mini Kit (QIAGEN, Inc., Valencia, CA, USA). Samples were pretreated without pre-lysis using the sample prep spin protocol. DNA was quantified by Nanodrop Spectrophotometer ND-1000 (Thermo Scientific, Wilmington, DE, USA) for both protocols. 

### 2.3. PCR Protocols

cPCR: HgapF and HgapR primers described by Aghazadeh et al. [1] were used for conventional PCR screening for DCH (Table 2). A master mix of 5 µL buffer, 0.5 µL dNTPs, 0.5 µL platinum SuperFi II DNA polymerase, 1 µL 10 µM forward primer, 1 µL 10 µM reverse primer, 15 µL water, and 2 µL 1–10 ng/µL sample DNA was used for each reaction. The following cycling conditions were used: 95 °C for 3 min, 35 cycles of 95 °C for 30 s, 60 °C for 30 s, 72 °C for 2 min, followed by 72 °C for 5 min, and 4 °C. No-template (molecular-grade water) and positive controls (DCH-positive whole blood derived DNA) were included in all cPCR assays. Products were resolved using 1.5% agarose gel electrophoresis.

qPCR: All samples tested by conventional PCR were also tested using qPCR. Primers published by Lanave et al. [16] were utilized (qPCR primers, Table 2). A PCR master mix of 12.5 µL iTaq Universal Probe Supermix (Bio-Rad, Hercules, CA, USA), DNA polymerase, 4.75 µL water, 1.25 µL 10 µM forward primer, 1.25 µL 10 µM reverse primer, 0.25 µL probe, and 5 µL 1–10 ng/µL sample DNA was added into each well. Samples were run with a CFX Connect Real-time PCR Detection System (Bio-Rad, Hercules, CA, USA) using the following cycling conditions: 93 °C for 3 min, 45 cycles of 95 °C for 10 s, and 60 °C for 30 s. DNA samples were run in triplicate alongside no-template controls and a known DCH positive sample (gift of Patricia Pesavento, University of California, Davis, CA, USA). Samples were considered positive if two out of three replicates had a Ct of <38 and results could be verified with at least one replicate. The Australian cats had paired whole blood and plasma samples that were run to confirm our assay was effective on both sample types. 

### 2.4. Genome Sequencing of Positive Sample

A full length DCH genome from one U.S. cat was generated via overlapping PCR amplicons from Cir7F/HgapR and Cir5F/Cir7R primer sets (Table 2). A deletion at the 5′ end of the core protein was detected, and this was confirmed by generating additional sequences using Cir7F/Cir1R and Cir7F/Cir5R primer sets. Partial DCH genomes from three Australian cats were amplified using Cir7F/HgapR, Cir7F/Cir1R, and Cir7F/Cir5R primer sets [1]. Products were amplified using reaction protocol and cycling conditions described above. Samples were ligated into Pjet plasmids using the CloneJET PCR cloning kit protocol (Thermo Scientific, Wilmington, DE, USA). A master mix of 1 µL 10× ligase buffer, 1 µL ligase, 2.75 µL distilled water, and 0.25 µL Pjet 1.2 vector was used. 5 µL of the PCR product was added and the mix was left at room temperature for 30 min. A transformation mix of 50 µL XL2-Blue MRF *Escherichia coli* ultracompetent cells (Agilent technologies, Santa Clara, CA, USA) and 5 µL ligation product was incubated on ice for 10 min, and then incubated at 42 °C for 45 s followed by 2 min on ice. Two hundred fifty µL LB was added and incubated at 37 °C for 25–30 min. The cells were then plated on LB and ampicillin culture plates and incubated at 37 °C for 16–18 h. 

To confirm that colonies had incorporated the intended PCR fragment, individual colonies were selected for qPCR by adding PCR master mix with 5 µL taq polymerase, 4 µL water, and 1 µL Pjet primers and the following cycling conditions: activation of Kappa Taq at 95 °C for 3 min, followed by 35 cycles of denaturation 95 °C for 30 s, annealing at 60 °C for 30 s, and extension for 72 °C for 2 min, with a final annealing temperature at 72 °C for 5 min. Bacterial colonies that tested positive for DCH were inoculated in individual 6–10 mL cultures of lysogeny broth and ampicillin. Cultures were then grown overnight at 250 rpm and 37 °C. Each bacterial culture was centrifuged, and plasmids were purified using the DNA-Spin plasma purification kit (iNrTON Biotechnology, Sagimakgol-ro Joongwon-gu, Seongnam-Si, Korea). Using the manufacturer’s instructions, a total of 50 µL of plasmid was eluted. Plasmids were Sanger sequenced at Psomagen (Rockville, MD, USA) and analyzed using Geneious (Biomatters, Auckland, New Zealand). 

Sequence analysis (including relatedness of DNA and protein sequences) was carried out on the U.S. DCH consensus sequence and previously published DCH genomes using Uniprot Align [23]. We included a human HBV DNA sequence (Accession number U95551.1) and a hepadnavirus isolated from the tent-making bat (Accession number KC790378.1) in the analysis as outgroups. The first 50 amino acids of the PreS1/S2-HBs ORF were analyzed by Myristoylator [24] to determine the probability of N-terminal myristoylation, with the human HBV DNA sequence as a positive control.

## 3. Results

### 3.1. Molecular Detection of DCH 

**Figure 1:**
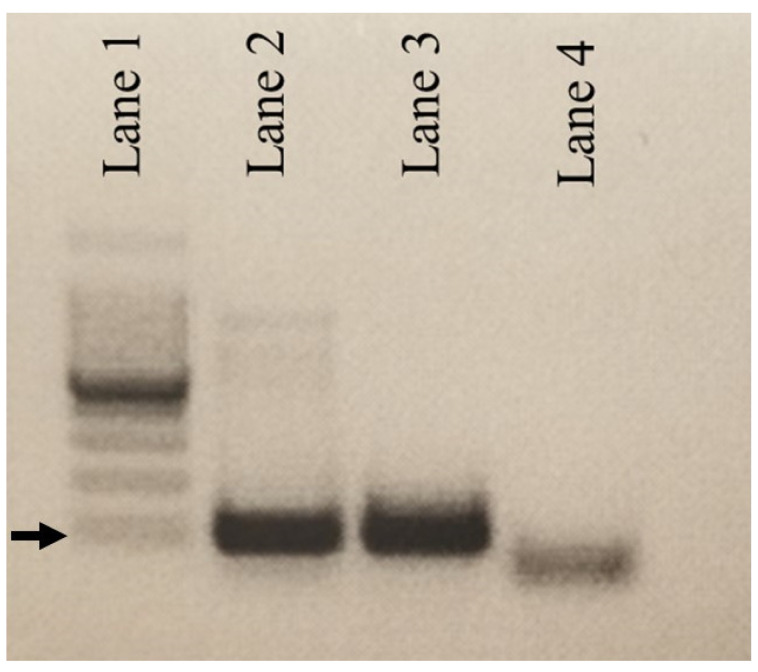
Gel electrophoresis products from cPCR in DNA isolated from a U.S. cat. DCH is identified by PCR from one cat from Ventura County, California. Lane 1 = 1 KB ladder; Lane 2 = PCR product from DNA extracted from the blood of a shelter cat from Ventura County, CA; Lane 3 = positive control provided from UC Davis; Lane 4 = Negative control (SPF cat DNA). Primers used are HgapF and HgapR with an expected size range of 200 BP. Arrow indicates approximately 200 bp.

A single sample among 496 cat samples from the USA tested positive for DCH on both cPCR (Figure 1) and qPCR, giving an estimated prevalence of infection of 0.2%. In contrast, 7 of 67 cat samples from Australia tested positive for DCH, giving a prevalence of 10.4%. Of the seven DCH positive Australian samples, four were positive on cPCR; three of these had strong bands and one a weak band on gel electrophoresis. The average Ct value for all four of these positive samples was 19.2, with the U.S. sample having an average Ct value of 17.8. The other three Australia samples, which were negative on cPCR, tested positive for DCH with Ct values averaging 35.8. 

A comparison of qPCR amplification of whole blood DNA extracts and plasma DNA extracts was carried out for two separate individuals. We found that DNA amplification was successful with both plasma DNA and whole blood DNA extracts. DNA extracted from whole blood was detected four cycles earlier than plasma, indicating an approximately 16x higher concentration in blood than plasma. 

### 3.2. DCH Genomic Analysis

We amplified the full length U.S. DCH genome using overlapping PCR amplicons, cloned them into plasmid backbones, and sequenced inserts using Sanger sequencing. The consensus genome sequence was assembled, and was aligned to the Sydney DCH isolate as a reference sequence (accession number MH307930) [1]. We found the U.S. DCH sequence to be 92.3% homologous to the Sydney DCH isolate.

**Figure 2:**
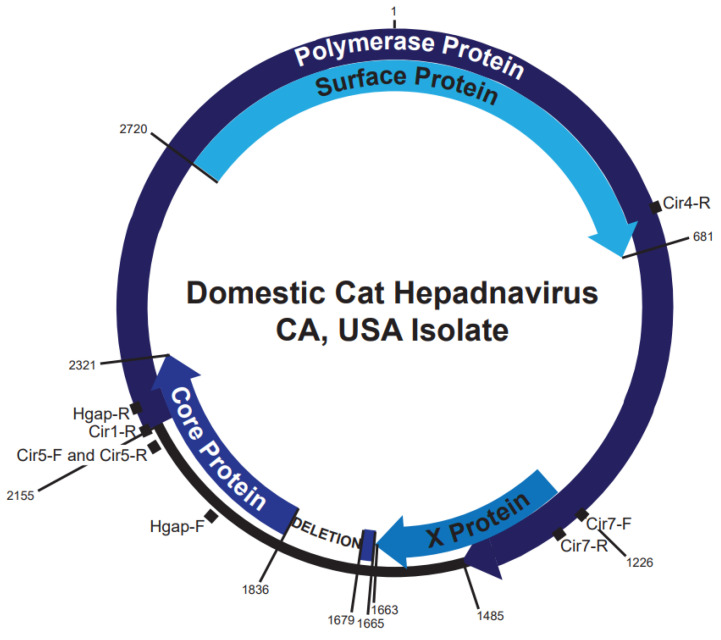
Whole genome DCH from a U.S. domestic cat. Overlapping clones were amplified from a cat from Ventura County, CA, USA. Sequences were aligned to the Sydney DCH reference sequence MH307930. A 157 bp deletion was observed in the core protein open reading frame between nucleotides 1679 and 1836. Multiple primer pairs and clones overlapping this region consistently detected the deletion. Nucleotide 1 represents a conserved EcoR1 site. Conventional PCR binding sites are indicated.

A 157 base-pair in-frame deletion within the core open reading frame was observed following multiple PCR amplifications (Figure 2). The deletion maintained the start codon of the putative pre-core protein and the first 5aa, followed by a deletion of 52aa, essentially leading to a 30aa N-terminal truncation of putative core protein. It is unclear whether this is still a functional capsid protein, though it preserves much of the assembly domain and the entire arginine-rich RNA/DNA binding domain. Nevertheless, this DNA sequence likely represents a replication-deficient genome as the deletion removes the predicted epsilon sequence, a necessary signal for viral RNA packaging and reverse transcription of the viral genome [25]. 

This deletion occurred near the putative DR1 region, i.e., the expected 5′ terminus of the double-stranded linear DNA (dslDNA) form described in hepadnaviruses, including human, duck, and woodchuck hepatitis B viruses [26]. This suggested that the deletion occurred as result of illegitimate recombination of dslDNA, which is known to occur upon nuclear import of dslDNA and formation of a defective covalently-closed circular DNA molecule [27,28]. Thus, this data suggests that DCH also produces dslDNA intermediates like other Hepadnaviruses, though this requires further confirmation in viral replication systems (e.g., cell culture models).

In comparison, three positive samples from Australia were amplified using the Cir7F/HgapR, Cir7F/Cir5R, and Cir7F/Cir1R primers and sequenced. Sanger Sequencing generated approximately 1000 bp of sequence, which matched with the original DCH reference genome (1). A gap in the core protein was also seen at nt1454–1687, which is in a different region than the gap found in the U.S. positive sample. 

**Figure 3:**
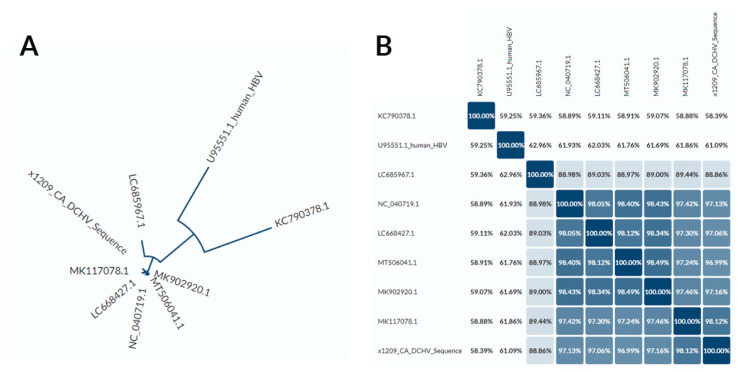
Phylogram (**A**) and identity matrix (**B**) based on whole DNA genome of DCH isolates, human HBV, and tent-making bat HBV. LC685967.1 = Japan isolate 1 (Rara); NC_040719.1 = Sydney isolate; LC668427.1 = Japan isolate 2; MT506041.1 = Thailand isolate; MK902920.1 = Malaysia isolate; MK117078.1 = Italy isolate. Alignment and figure generated using Uniprot Align [23].

We then compared the genome of the U.S. DCH isolate to those isolated in other countries (Figure 3) using Uniprot Align. We found that the closest sequence similarity was to an isolate from a cat in Italy. 

After comparing DNA sequence identity (Figure 3B), we found at least two different DCH genotypes (defined by >8% nucleotide difference): one represented by the isolate from Rara, Japan (Genbank Accession LC685967.1), and the other defined by the rest of the sequences, which were closely related. 

### 3.3. DCH Surface Protein Analysis

**Figure 4:**
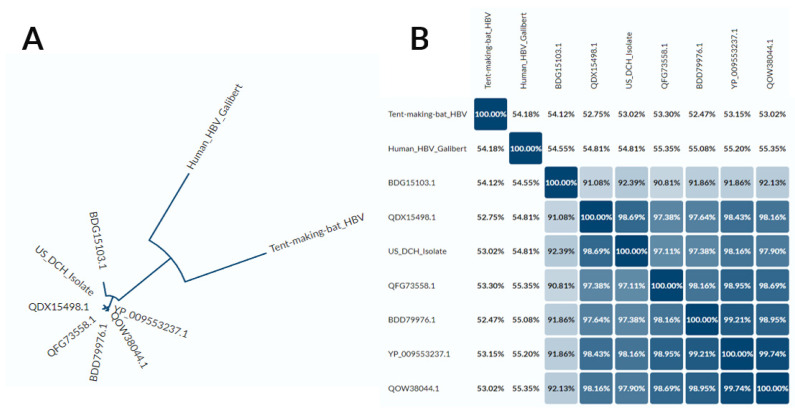
Phylogram (**A**) and identity matrix (**B**) based on the large surface protein sequence of DCH isolates, human HBV, and tent-making bat HBV. BDG15103.1 = Japan isolate 1 (Rara); YP_009553237.1 = Sydney isolate; BDD79976.1 = Japan isolate 2; QOW38044.1 = Thailand isolate; QFG73558.1 = Malaysia isolate; QDX15498.1 = Italy isolate. Analysis and figure generated using Uniprot Align [23].

Given the importance of the hepadnaviral large envelope protein in host tropism and viral entry, we analyzed in greater detail the amino acid sequence of the U.S. DCH isolate in the context of DNA sequences of the other DCH isolates, human HBV, and tent-making bat HBV. The phylogram (Figure 4A) and identity matrix (Figure 4B) based on the amino acid sequence of the large DCH surface protein largely reflected those generated using the whole DNA genome (Figure 4), with the two outgroups equally distant from the DCH sequences and the existence of two genotypes. 

**Figure 5:**
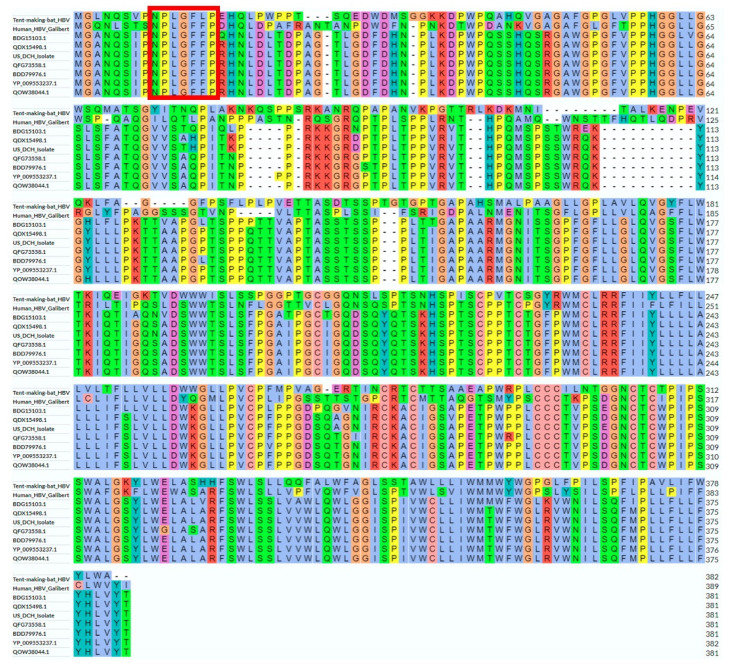
Sequence alignment of the whole large surface protein sequence of DCH isolates, human HBV, and tent-making bat HBV. Red rectangle represents the highly conserved domain necessary for NTCP binding. BDG15103.1 = Japan isolate 1 (Rara); YP_009553237.1 = Sydney isolate; BDD79976.1 = Japan isolate 2; QOW38044.1 = Thailand isolate; QFG73558.1 = Malaysia isolate; QDX15498.1 = Italy isolate. Analysis and figure generated using Uniprot Align [23].

Despite the significant differences and limited sequence identity to other hepadnaviruses, we found important conserved regions in the DCH large surface antigen sequence (Figure 5), namely those involved in binding to sodium taurocholate cotransporting polypeptide (NTCP), the cellular receptor for human HBV [29,30]. Firstly, the NTCP-binding domain 9-NPLGFFP-15 is completely conserved in all known human and primate HBV sequences, and mostly conserved in the NTCP-binding tent-making HBV [31]. Importantly, we show here that all known DCH sequences contain the 9-NPLGFFP-15 domain. Further, using the motif prediction software Myristoylator [24], all DCH sequences were highly probable (>90%) to be subject to N-terminal N-myristoylation, a necessary feature of human HBV to bind NTCP [32]. 

## 4. Discussion

We found a very low prevalence of DCH among the general population of cats located in the U.S. relative to reported incidence in Australia and Italy. A previous study linking DCH to feline chronic hepatitis and HCC, consistent with other hepadnaviruses, included nine such cases from the U.S., of which >40% contained DCH sequences [17,33]. Notably, screening of 56 cat liver samples obtained from cats with hepatic lymphoma in this study did not identify DCH genomic material.

It is unclear why the prevalence detected in this study of multiple U.S. cat populations was so low. Differences in breeding procedure, veterinary practices, or feline behavior between the different regions may play a role in the prevalence of chronic DCH infections. While a significant number of U.S. cats tested were from shelters, a total of 95 samples (84 from accessions to the CSU hematopathology laboratory and 11 from the CSU VDL necropsy service) were representative of typical companion animals. Maintenance of hepadnavirus infections in a population generally requires ongoing infections between hosts, usually vertical transmission during birth. Both viral load and age at exposure determine the outcome of hepadnavirus infections [34]. Future observational studies should be carried out on mother/kit pairs to determine if high DCH DNA loads is a risk factor for DCH transmission. 

We considered the role of co-infection with FIV as a possible driver of lower prevalence of DCH. Indeed, the initial discovery of DCH was made in immunocompromised individuals [1]. However, our study did not support this hypothesis: plasma from cats from shelters in the midwestern U.S. represented a population with high levels of FIV [35], cats from a household with nearly 50% FeLV progressive infections [20] were tested, and no DCH positive animals were identified. 

Our sequence analysis showed that the DCH DNA genome detected in the U.S. sample is likely replication-incompetent and probably represents defective cccDNA (though there is a possible chance of reversion to infectious forms) [36]. Given the absence of an epsilon sequence, the RNA molecule derived from this sequence should theoretically not have been encapsulated and reverse-transcribed. Thus, this sequence is not likely to have come from a virion containing DNA. A possibility is that the sequence is derived from circulating hepatocyte-derived DNA, which has previously been detected in the blood and urine [37,38,39]. Moreover, replication-defective cccDNA molecules are known to persist in the liver, particularly after acute infection [40]. Thus, it is possible that this DNA sequence derives from leakage of intrahepatic viral DNA, resultant from either a current or past DCH infection. 

Regardless, the nature of the deletion suggests similar viral intermediates as other hepadnaviruses (e.g., formation of double-stranded linear forms that can generate defective cccDNA molecules). Further in vitro replication experiments could be done to experimentally confirm our results. 

Whole genome sequence analysis supported the co-evolution of DCH with the host, rather than crossing a host species barrier between bats and cats. The DCH genome sequenced here is slightly more distant from bat HBV compared to the human HBV isolate, suggesting that the origin of DCH was unlikely to be due to species jumping from bats to cats. Furthermore, we found all DCH sequences outside of Japan represented a distinct genotype, which suggests relatively recent DCH spread and/or high levels of population inter-mixing outside of Japan. This is consistent with the narrow host range of all other known hepadnaviruses. However, through detailed analysis of the surface proteins, we show here for the first time that it is highly likely that DCH uses NTCP as an entry receptor. Further experiments are needed to confirm the entry receptor.

It is important to note that circulating DCH DNA is likely not a sensitive technique to detect chronic infection and likely underestimates prevalence. Throughout a human chronic HBV infection, viral loads can vary enormously (ranging between >10^9^ to 0 viral copies per mL of blood) and this is likely true also for DCH infection. The current sensitivity of our assay is not known and further analysis including absolute quantification is needed. If the natural history of chronic DCH infection reflects that of other hepadnaviruses, then ELISAs to detect the surface protein (for current DCH infection) and anti-core antibodies (for previous exposure to DCH) would be ideal to determine prevalence more accurately in cat populations. Further investigation of DCH antibody prevalence in the U.S. domestic cat population would provide information about U.S. domestic cat exposure to DCH and its possible impacts to cat health. 

In conclusion, we identify low prevalence of DCH in the U.S. compared to other sampled countries using both conventional and qPCR screening assays. This work has revealed the likely replication mechanism, via the presence of a deletion in U.S. isolate, and implicates NTCP as the likely entry receptor. The distinction of DCH from all known hepadnaviruses is additional evidence for the greater extant diversity of non-human hepadnaviruses than previously recognized. 

## Figures and Tables

**Table 1:** Conventional and qPCR detection of DCH in cat samples reveals one positive sample out of 496 samples from diverse U.S. populations and seven positive samples out of 67 samples from Australia. WB = whole blood. One positive sample identified from Ventura County shelters in California was cloned for complete genome sequencing as described below. Of 67 samples from paired whole blood and plasma from Australian domestic cat samples, 7 were identified and 3 of these were also sequenced. Conventional PCR was executed via HGAP primers as described by Aghazadeh et al. [1]. qPCR was performed using FHBV primers and protocols as described by Lanave et al. [16].

Sampling Cohort	Reference	Tissue Sampled	Positive Conventional PCR Analyses/Total Analyses	Positive qPCR Confirmatory Analyses/Total Analyses	DCH Positive Samples/Total Samples	Positive (%)
Multi-cat household	Powers, Jordan, et al., Journal of Virology [20]	WB DNA	0/57	0/57	0/57	0
Cats presented to shelters in Colorado, California, Florida	Carver, Scott, et al., Ecological Applications [19]	WB DNA	1/286	1/286	1/286	0.35
CSU Hematopathology Laboratory	Gift of Anne Avery, Laboratory Director	Hepatic DNA	0/84	0/84	0/84	0
CSU VDL necropsy	CSU Diagnostic Laboratory	Hepatic DNA	0/11	0/11	0/11	0
Memphis animal shelters and households	Ledesma-Felicano, Carmen, et al., Retrovirology [21]	Plasma	0/58	0/58	0/58	0
TOTAL US			496	496	1/496	0.2
University of Sydney, Sydney Australia	Ledesma-Feliciano, Carmen, et al., Viruses [22]	WB DNA/Plasma	4/67	7/67	7/67	10.4

**Table 2:** PCR primers used in this study. All the primer sets listed above were used in this study for PCR product analysis. Primers from [1,16].

qPCR Primers	Sequence 5′–3′	Binding Site
FHBV F	CGTCATCATGGGTTTAGGAA	Nt 458→478, surface and polymerase proteins
HBV-R	TCCATATAAGCAAACACCATACAAT	Nt 589→565, surface and polymerase proteins
FHBV P	[FAM]TCCTCCTAACCATTGAAGCCAGACTACT [BHQ]	Nt 528→555, surface and polymerase proteins
**Conventional PCR Primers**	**Sequence 5′–3′**	**Binding Site**
Cir 5f	TTGGCACCTGGATTCGCA	Nt 2113→2130, core protein
Cir 4R	AGATGTTCCACACTCTTAGCC	Nt 625→605, surface and polymerase proteins
Cir 7R	CGTAGACGAAGGACACGTC	Nt 1281→1262, polymerase and X proteins
Cir 7F	CCATCGATTTACACACTTCCCA	Nt 1205→1226, polymerase protein
Cir 5R	TGCGAATCCAGGTGCCAA	Nt 2130→2113, core protein
Cir 1R	ATAACCGTATGCTCCGGAAG	Nt 2194→2175, core and polymerase proteins
Hgap F	GTGCTCTGATAACCGTATGCTC	Nt 1945→1968, core protein
Hgap R	CTAGAATGGCTACATGGGAG	Nt 2203→2181, core and polymerase proteins

## Data Availability

Viral sequence data accession number OP094657.
